# An unfolded protein response (UPR)-signature regulated by the NFKB–miR-29b/c axis fosters tumor aggressiveness and poor survival in bladder cancer

**DOI:** 10.3389/fmolb.2025.1542650

**Published:** 2025-02-14

**Authors:** Jian Zhang, Xiaosong Fan, Yu Chen, Yichao Han, Weixing Yu, Shaolin Zhang, Bicheng Yang, Junlong Zhang, Yanling Chen

**Affiliations:** ^1^ Department of Urology, Shangyu People’s Hospital of Shaoxing, Shaoxing University, Shaoxing, Zhejiang, China; ^2^ Zhejiang Hisoar Pharmaceutical Co Ltd., Hangzhou, Zhejiang, China; ^3^ Department of Urology, The First Affiliated Hospital, Sun Yat-sen University, Guangzhou, Guangdong, China; ^4^ Department of Neurosurgery, The First Affiliated Hospital of Wannan Medical College (Yijishan Hospital of Wannan Medical College), Wuhu, Anhui, China; ^5^ Digestive Endoscopy Center, The First Affiliated Hospital of Wannan Medical College (Yijishan Hospital of Wannan Medical College), Wuhu, Anhui, China

**Keywords:** bladder cancer, ER stress, UPR, inflammation, NFkB, miR-29b/c

## Abstract

**Background:**

Bladder cancer continues to pose a substantial global health challenge, marked by a high mortality rate despite advances in treatment options. Therefore, in-depth understanding of molecular mechanisms related to disease onset, progression, and patient survival is of utmost importance in bladder cancer research. Here, we aimed to investigate the underlying mechanisms using a stringent differential expression and survival analyses-based pipeline.

**Methods:**

Gene and miRNA expression data from TCGA and NCBI GEO databases were analyzed. Differentially expressed genes between normal vs tumor, among tumor aggressiveness groups and between early vs advanced stage were identified using Student's t-test and ANOVA. Kaplan-Meier survival analyses were conducted using R. Functional annotation, miRNA target and transcription factor prediction, network construction, random walk analysis and gene set enrichment analyses were performed using DAVID, miRDIP, TransmiR, Cytoscape, Java and GSEA respectively.

**Results:**

We identified elevated endoplasmic reticulum (ER) stress response as key culprit, as an eight-gene unfolded protein response (UPR)-related gene signature (UPR-GS) drives aggressive disease and poor survival in bladder cancer patients. This elevated UPR-GS is linked to the downregulation of two miRNAs from the miR-29 family (miR-29b-2-5p and miR-29c-5p), which can limit UPR-driven tumor aggressiveness and improve patient survival. At further upstream, the inflammation-related NFKB transcription factor inhibits miR-29b/c expression, driving UPR-related tumor progression and determining poor survival in bladder cancer patients.

**Conclusion:**

These findings highlight that the aberrantly activated UPR, regulated by the NFKB-miR-29b/c axis, plays a crucial role in tumor aggressiveness and disease progression in bladder cancer, highlighting potential targets for therapeutic interventions and prognostic markers in bladder cancer management.

## 1 Background

Cancer, spanning a broad spectrum of malignancies, continually pushes the boundaries of medical research (Soerjomataram and Bray, 2021). Bladder cancer, affecting the urinary tract, ranks as the tenth most prevalent tumor globally, with more than half a million new cases and 200,000 deaths reported annually (Bray et al., 2018). Most bladder cancer patients present a non-muscle invasive phenotype, managed through local treatments or surveillance (Wu, 2005; Chang et al., 2017). Treatment for non-metastatic patients typically involves surgical resection or a combination of local resection, radiation, and chemotherapy (Chang et al., 2017). Conversely, platinum-based combination therapies are mainly used for unresectable or metastatic bladder cancers. Despite these efforts, the median overall survival remains low, at approximately 14 months for bladder cancer patients (von der Maase et al., 2000; Sternberg et al., 2006). Recent advances in immune checkpoint blockade have shown promise in extending survival for some bladder cancer patients, although the response rate is limited (Bellmunt et al., 2017).

It is crucial to recognize our current gaps in understanding bladder cancer at the molecular level. These limitations significantly hinder our ability to effectively treat these tumors, leading to less favorable prognoses. Therefore, there is an urgent need to identify targetable molecular mechanisms associated with various aspects of bladder cancer, including disease onset, progression, tumor aggressiveness, and patient outcomes (Soria et al., 2019; Flores Monar et al., 2023). These insights may pave the way for developing more precise diagnostic tools and targeted therapeutic approaches, ultimately improving the prognosis and survival rates for bladder cancer patients.

Inflammation is a crucial defense mechanism against cellular or organ damage, where the immune system mobilizes responses to neutralize harmful stimuli and initiate healing processes. However, prolonged or excessive inflammation has been implicated in promoting carcinogenesis and tumor progression, activating a cascade of inflammatory mediators and signals (Virchow, 1989; Mantovani et al., 2008). Recent research has specifically implicated inflammation in the initiation and advancement of bladder cancer. Chronic inflammation, whether systemic or localized, has been associated with an elevated risk of bladder cancer development. Furthermore, oncogenic alterations can create a chronically inflamed microenvironment within bladder tissues, exerting multifaceted tumor-promoting effects such as enhanced cell proliferation, angiogenesis, invasion, and metastasis (Kim and Ku, 2016; Sui et al., 2017). The unfolded protein response (UPR) is a critical cellular pathway that responds to endoplasmic reticulum (ER) stress, a condition commonly encountered in cancer cells due to proliferative burden-related high protein synthesis demands (Walter and Ron, 2011). The UPR can facilitate tumor cell survival and adaptation by promoting protein folding, enhancing ER capacity, and mitigating stress-induced damage, enabling cancer cells to thrive in adverse microenvironments characterized by nutrient deprivation, hypoxia, oxidative stress, and/or inflammation (Jain, 2017). Despite this, the molecular mechanisms entailing inflammation and UPR in bladder cancer are not well established. Therefore, understanding these molecular mechanisms is crucial for developing targeted therapies that exploit the vulnerabilities in bladder cancer.

The emergence of advanced expression profiling techniques, along with the availability of extensive datasets, has empowered scientists to delve swiftly into the molecular mechanisms underlying diseases. This progress has been instrumental in advancing our understanding of disease pathogenesis and identifying potential targets for diagnosis, prognosis, and therapeutic interventions (Tirosh and Suvà, 2019; Sheng et al., 2020). Here, we utilized a rigorous approach combining differential expression and survival analyses to explore the underlying molecular mechanisms in bladder cancer using the available online expression profiling data. We identified elevated ER stress response as a key driver of the disease and constructed an eight-gene UPR-related gene signature (UPR-GS) associated with an aggressive disease state and poor survival in bladder cancer patients. Notably, the upregulation of UPR-GS was linked to the downregulation of miR-29b-2-5p and miR-29c-5p, which have the potential to restrain UPR-driven tumor aggressiveness and contribute to favorable survival outcomes in bladder cancer. Inflammation-related NFKB transcription factor rescues UPR-GS from miRNA-mediated suppression by directly inhibiting the expression of miR-29b/c upstream, thereby promoting UPR-related tumor aggressiveness and contributing to poor survival in bladder cancer patients. These findings underscore the pivotal role of the aberrantly activated UPR, governed by the NFKB-miR-29b/c axis, in driving disease progression in bladder cancer and highlight that restoring miR-29b/c expression offers a promising strategy to limit UPR and mitigate tumor aggressiveness in bladder cancer patients.

## 2 Methods

### 2.1 Data retrieval

Bladder cancer patients’ gene expression (mRNAseq_Preprocess (MD5)) and miRNA expression miRseq preprocess (MD5)) data were retrieved from the online Cancer Genome Atlas (TCGA) database along with the clinical observations (Clinical_Pick_Tier1 (MD5)) file from Broad GDAC Firehose: https://gdac.broadinstitute.org/. In addition, expression data of bladder cancer patients were retrieved from the GEO database for the following datasets: GSE120736 and GSE13507 (Kim et al., 2010; Lee et al., 2010). Briefly, a pre-processed “Series matrix file” (containing clinical observations and expression values) was downloaded along with the associated sequencing platform GPL file (containing a list of Probe IDs with associated gene symbols). Expression data were aligned with gene symbols and analyzed against clinical observations: normal vs. tumor, tumor stage, tumor grade, muscle invasiveness, disease progression, and survival analysis. Data on the CRISPR screen-based chronos dependency score of genes in UPR-GS were retrieved from the Dep-Map portal (https://depmap.org/portal/) for bladder cancer cell lines. Scores were then averaged to obtain an overall dependency score for the UPR-GS pathway. A negative dependency score indicates the relative essentiality of a gene for a given cell line, whereas a score of 0 indicates that the gene is non-essential.

### 2.2 Patient tumor aggressiveness classification

The epithelial-to-mesenchymal transition (EMT) has been established as a prime marker for tumor aggressiveness and metastasis (Yang and Weinberg, 2008; Voulgari and Pintzas, 2009; Sethi et al., 2011; Andergassen et al., 2018). The Hallmark_EMT gene set was downloaded from the Gene Set Enrichment Analysis (GSEA) portal https://www.gsea-msigdb.org/ (Supplementary Table S1), and Z-scores of all the 200 genes in this signature for each patient were calculated (Supplementary Table S2). Later, Z-scores were summed to calculate the tumor aggressiveness score for each patient. Lastly, patients were sorted according to their tumor aggressiveness score and were divided into three equal groups: low, intermediate, and high.

### 2.3 Survival analyses

Kaplan–Meier survival curves were generated for all the genes in the TCGA database using R. Patients without available survival time or event data were excluded from the corresponding patient groups. All separations were made according to the better cutoff threshold. The significance of the differences in survival between the two groups was calculated by the Log-rank (Mantel–Cox) test. Significance cutoffs were taken as p < 0.05.

### 2.4 Identification of oncogenic targets

The mRNA expression of all the genes was compared 1) between normal and tumor tissue, 2) among tumor aggressiveness groups, and 3) between early- and advanced-stage tumor tissues from the TCGA database. A false discovery rate (FDR) significance cutoff of < 0.05 was used in these analyses. In addition, survival analysis was performed for all the genes. Later, the intersecting genes 1) upregulated in tumor tissues compared to normal tissues, 2) successively upregulated from low to high aggressiveness group, 3) upregulated in advanced-stage tumors compared to early ones, and 4) associated with poor survival were considered as oncogenic targets in bladder cancer.

### 2.5 Pathway enrichment analysis

Pathway enrichment analysis was performed using the freely available online DAVID functional annotation tool: https://david.ncifcrf.gov/summary.jsp. Briefly, a list of identified oncogenic targets was uploaded to the DAVID platform to identify the associated KEGG pathways, GO: Biological process, GO: Molecular function, and GO: Cellular compartment. A significance cutoff of p <0.05 was used.

### 2.6 Network construction and random walk analysis

A list of identified oncogenic targets was uploaded to the String database: https://cn.string-db.org/. A file consisting of interactions was downloaded and uploaded to Cytoscape 3.7.1 to construct the final network. Random walk analysis was performed using Java code. Briefly, an equal amount of energy was introduced from each node in the network. N iterations were performed until energy was distributed in the network and reached a steady-state level. The energy retained by each node after reaching the steady-state level was considered a random walk score.

### 2.7 Gene set enrichment analysis (GSEA)

Tumor aggressiveness scores, UPR-GS scores, and miR-GS scores were calculated by summing the expression Z-scores of each gene in the given gene set for each patient in the TCGA database (Supplementary Table S2). Patients were divided according to 1) tumor stage (I and II vs. III and IV), 2) tumor aggressiveness scores (low vs. high: patients were sorted in ascending order based on calculated scores and divided into two groups of “low” and “high” with an equal number of patients in each group), 3) UPR-GS scores (low vs. high), or 4) miR-GS scores (low vs. high). GSEA was performed using gene sets related to 1) ER stress, 2) bladder cancer, 3) NFKB and MYC transcription factor (TF), 4) inflammation, 5) proliferation, 6) cancer progression, and 7) metabolic adaptation, downloaded from the GSEA website: http://software.broadinstitute.org/gsea/index.jsp. A list of gene sets used is provided in Supplementary Table S1.

### 2.8 miRNA-target and transcription factor prediction analyses

miRNA-target prediction analysis was performed using a freely available miRDIP tool (https://ophid.utoronto.ca/mirDIP/), where miRNAs from miR-29-GS and genes from UPR-GS were uploaded, and a predicted interaction list was downloaded. TF prediction for mRNAs was performed at the freely available hTFtarget database: http://bioinfo.life.hust.edu.cn/hTFtarget#!/, where genes from UPR-GS were uploaded, and a list of predicted TFs was downloaded. TF prediction for miRNAs was performed at the TransmiR database v.2.0: http://www.cuilab.cn/transmir, where TFs were individually searched for miRNAs from miR-29-GS, and results were downloaded.

### 2.9 Statistical analysis

In order to compare data between the two groups, a Student’s t-test was performed. One-way ANOVA was performed to compare data among more than two groups. For correlation analysis, the Pearson correlation coefficient was calculated. Bar graphs, dot plots, forest plots, and survival plots were made using GraphPad Prism v6: https://www.graphpad.com. Illustrations were made in Biorender: https://www.biorender.com. Venn diagrams were made online at http://bioinformatics.psb.ugent.be/webtools/Venn.

## 3 Results

### 3.1 ER stress response is associated with disease onset and progression, tumor aggressiveness, and survival in bladder cancer

**FIGURE 1:**
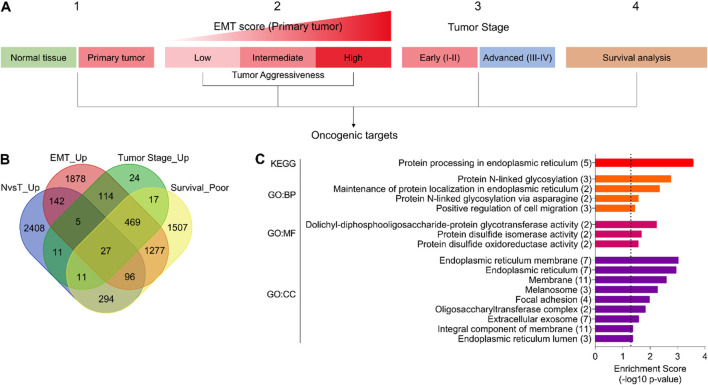
The ER stress response is associated with disease onset and progression, tumor aggressiveness, and survival in bladder cancer. **(A)** An illustration showing the analysis pipeline used to identify oncogenic targets in bladder cancer from the TCGA database. Briefly, gene expression was compared (1) between normal and tumor tissues, (2) among EMT score-based tumor aggressiveness groups (low, intermediate, high), and (3) between early- and advanced-stage tumors. In addition, (4) survival association was also checked. **(B)** A Venn diagram showing a number of oncogenic targets identified through the pipeline in **(A)**. **(C)** A bar graph showing the results of pathway enrichment analysis using common oncogenic targets identified in **(A, B)**. EMT, epithelial-to-mesenchymal transition; N, normal; T, tumor; GO, gene ontology; BP, biological process; MF, molecular function; CC, cellular compartment.

In order to identify the oncogenic targets associated with disease onset and progression, tumor aggressiveness, and survival, we downloaded the gene expression data of bladder cancer patients from the TCGA database and performed differential expression analysis (1) between normal and tumor tissue, (2) among low, intermediate, and highly aggressive tumor groups (based on EMT scores) (For details, see the *Materials and Methods* section), and (3) between early- and advanced-stage tumors. In addition, the Kaplan–Meier survival association was also estimated for all the genes available in the TCGA database (Figure 1A). As a result of these analyses, we found 27 genes that were (1) upregulated in tumors compared to normal tissue, (2) successively upregulated from low to highly aggressive tumor groups, (3) upregulated in advanced-stage tumors compared to early-stage ones, and (4) associated with poor survival in bladder cancer patients (Figure 1B). Next, we performed pathway enrichment analyses, which suggested that these genes are associated with protein processing in the ER (Figure 1C). The proliferative burden during tumor onset and progression often puts protein folding stress on the ER to meet the ever-increasing demands. In turn, cancer cells adapt different mechanisms to cope with ER stress, particularly UPR (Wang and Kaufman, 2014; Gsottberger et al., 2023). In order to validate this, we performed GSEA using different gene sets associated with protein folding in the ER and ER stress response and found that these gene sets are enriched in patients with advanced-stage tumors compared to early-stage ones (Supplementary Figures S1A–F), confirming that the ER stress response is indeed associated with aggressive disease state in bladder cancer patients.

### 3.2 A UPR-related gene signature determines aggressive disease state and poor survival in bladder cancer

**FIGURE 2:**
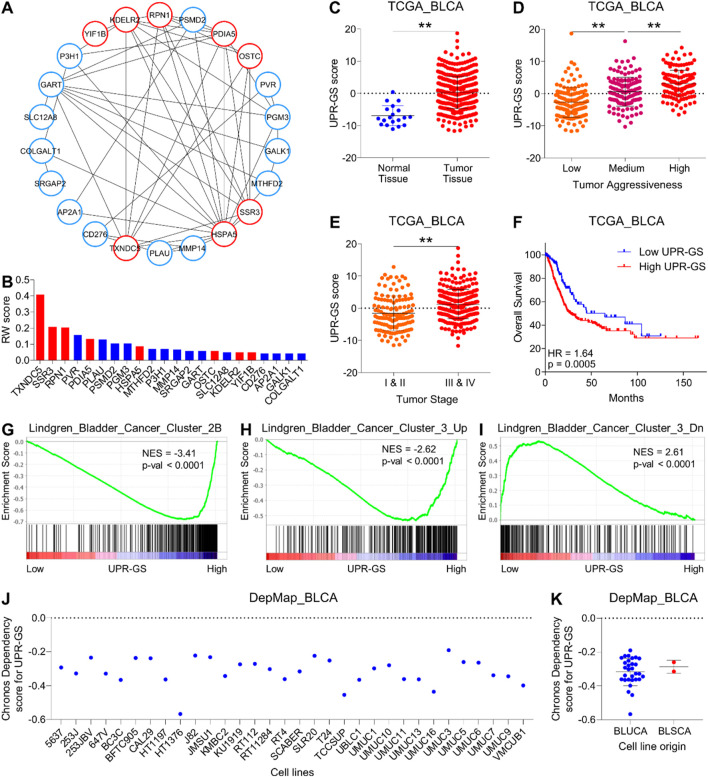
A UPR-related gene signature determines aggressive disease state and poor survival in bladder cancer. **(A)** An illustration showing the interaction network of genes associated with significant terms identified through pathway enrichment analysis in Figure 1C. Nodes related to UPR are highlighted in red. **(B)** A bar graph showing the results of a random walk analysis of the interaction network from **(A)**. Genes are sorted from high to low random walk (RW) scores, and genes associated with UPR are highlighted in red. **(C)** A dot plot showing changes in UPR-GS score between normal and tumor tissues in bladder cancer patients from the TCGA database. **(D)** A dot plot showing changes in UPR-GS scores among low, medium, and highly aggressive tumor groups in bladder cancer patients from the TCGA database. **(E)** A dot plot showing changes in UPR-GS scores between early- and advanced-stage tumors in bladder cancer patients from the TCGA database. **(F)** A Kaplan–Meier survival plot showing overall survival analysis based on low and high UPR-GS scores in bladder cancer patients from the TCGA database. **(G–I)** GSEA showing the enrichment of bladder cancer-related gene sets, Lindgren_Bladder_Cancer_Cluster_2B **(G)**, Lindgren_Bladder_Cancer_Cluster_3_Up **(H)**, and Lindgren_Bladder_Cancer_Cluster_3_Dn **(I)** between low and high UPR-GS score expressing tumors in bladder cancer patients from the TCGA database. **(J)** A dot plot showing the CRISPR screen-based chronos dependency scores of UPR-GS for 32 bladder cancer cell lines. **(K)** A dot plot summarizing the CRISPR screen-based chronos dependency scores of UPR-GS for cell lines representing bladder urothelial and squamous cell carcinoma. BLCA, bladder cancer; BLUCA, bladder urothelial carcinoma; BLSCA, bladder squamous cell carcinoma; Up, upregulated; Dn, downregulated; NES, normalized enrichment score. **, p < 0.01.

In order to identify key oncogenic hubs, we first constructed a network using the String database and found that 22 genes (of 27) make an interacting network. Notably, eight (thioredoxin domain containing 5 (TXNDC5), signal sequence receptor subunit gamma (SSR3), ribophorin I (RPN1), protein disulfide-isomerase family A member 5 (PDIA5), heat shock protein family A member 5 (HSPA5), oligosaccharyltransferase complex subunit (OSTC), KDEL ER protein retention receptor 2 (KDELR2), and Yip1 interacting factor homolog B (YIF1B)) of these 22 genes are directly associated with UPR (Figure 2A). In the second step, we performed random walk analysis on this network and found that UPR-related genes relatively retain more energy when random walk reaches a steady state (Figure 2B) (For details, see the *Materials and Methods* section), suggesting that this UPR-related gene signature (UPR-GS) serves as a key hub in the oncogenic network of bladder cancer.

As expected, this UPR-GS is 1) upregulated in tumors compared to normal tissue (Figure 2C; Supplementary Figure S2A), (2) successively upregulated from low to high aggressive tumor groups (Figure 2D; Supplementary Figure S2A), (3) upregulated in advanced-stage tumors compared to early-stage ones (Figure 2E; Supplementary Figure S2A), and (4) associated with poor survival in bladder cancer patients (Figure 2F; Supplementary Figure S2B). In addition, genes whose expression is specifically upregulated in Cluster IIB of bladder cancer are enriched in patients with tumors having high UPR-GS scores compared to those having low UPR-GS scores (Figure 2G). Furthermore, genes whose expression is specifically upregulated in Cluster III of bladder cancer are enriched in patients with tumors having high UPR-GS scores compared to those having low UPR-GS scores (Figure 2H), whereas genes whose expression is specifically downregulated in Cluster III of bladder cancer are enriched in patients with tumors having high UPR-GS scores compared to those having low UPR-GS scores (Figure 2I), confirming that UPR is indeed involved in tumor aggressiveness in bladder cancer. Notably, CRISPR screen-based data from 32 bladder cancer cell lines also suggested that all were moderately dependent on UPR-GS for proliferation and survival (Figures 2J, K), further affirming the importance of UPR-GS in bladder cancer.

**FIGURE 3:**
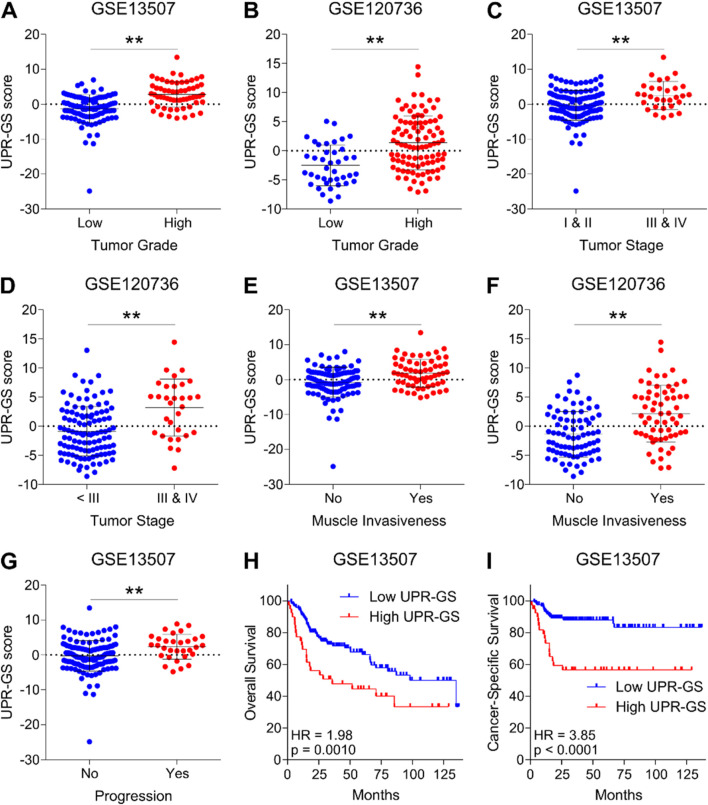
UPR-GS is associated with an aggressive disease state and poor survival in independent datasets of bladder cancer. **(A, B)** Dot plots showing changes in UPR-GS scores between low- and high-grade tumors in bladder cancer patients from GSE13507 **(A)** and GSE120736 **(B)**. **(C, D)** Dot plots showing changes in UPR-GS scores between early- and advanced-stage tumors in bladder cancer patients from GSE13507 **(C)** and GSE120736 **(D)**. **(E, F)** Dot plots showing changes in UPR-GS scores between non-muscle invasive and muscle invasive subtypes of tumors in bladder cancer patients from GSE13507 **(E)** and GSE120736 **(F)**. **(G)** A dot plot showing changes in UPR-GS scores with tumor progression in bladder cancer patients from GSE13507. **(H, I)** Kaplan–Meier survival plots showing overall survival **(H)** and cancer-specific survival **(I)** analysis based on low and high UPR-GS scores in bladder cancer patients from the GSE13507 dataset. **: *p* < 0.01.

Validating our findings, a high UPR-GS score is associated with high-grade tumors compared to low-grade ones in bladder cancer patients from GSE13507 (Figure 3A) and GSE120736 (Figure 3B). A high UPR-GS score is also associated with advanced-stage tumors compared to early-stage ones in bladder cancer patients from GSE13507 (Figure 3C) and GSE120736 (Figure 3D). A high UPR-GS score is associated with muscle-invasive tumors compared to non-invasive ones in bladder cancer patients from GSE13507 (Figure 3E) and GSE120736 (Figure 3F). A high UPR-GS score is associated with disease progression in bladder cancer patients from GSE13507 (Figure 3G). Lastly, a high UPR-GS score is associated with poor overall survival (Figure 3H) and poor cancer-specific survival (Figure 3I) in bladder cancer patients from GSE13507. Overall, these findings confirm that an aberrantly activated UPR determines an aggressive disease state and poor survival in bladder cancer patients.

### 3.3 miR-29 family miRNAs limit UPR-driven tumor aggressiveness and determine good survival in bladder cancer

**FIGURE 4:**
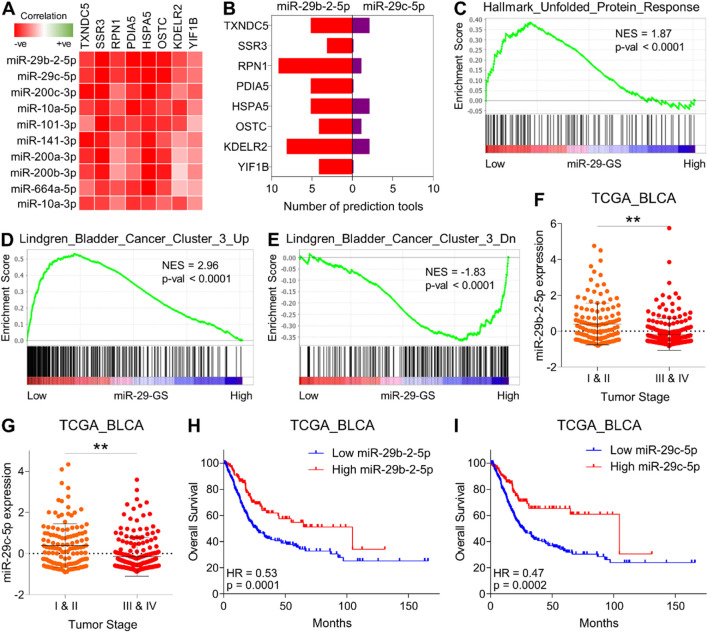
miR-29 family miRNAs limit UPR-driven tumor aggressiveness and determine good survival in bladder cancer. **(A)** A heatmap showing the correlation between genes from UPR-GS and the top ten miRNAs negatively correlated with these genes. **(B)** A bar graph showing miRNA-target prediction results for miRNAs (miR-29-2-5p and miR-29c-5p) against genes from UPR-GS. **(C–E)** GSEA showing the enrichment of Hallmark_Unfolded_Protein_Response **(C)**, Lindgren_Bladder_Cancer_Cluster_3_Up **(D)**, and Lindgren_Bladder_Cancer_Cluster_3_Dn **(E)** between low and high miR-29-GS score-expressing tumors in bladder cancer patients from the TCGA database. **(F, G)** Dot plots showing changes in miR-29b-2-5p **(F)** and miR-29c-5p **(G)** between early- and advanced-stage tumors in bladder cancer patients from the TCGA database. **(H, I)** Kaplan–Meier survival plots showing overall survival analysis based on low and high miR-29b-2-5p **(H)** and miR-29c-5p **(I)** expression in bladder cancer patients from the TCGA database. BLCA, bladder cancer; Up, upregulated; Dn, downregulated; NES, normalized enrichment score. **: p < 0.01.

Next, we aimed to determine how this UPR-GS is upregulated during the course of tumor progression in bladder cancer. MicroRNAs (miRNAs) are short non-coding RNA molecules that play a crucial role in post-transcriptional regulation of gene expression. They exert their regulatory functions by binding to specific messenger RNA (mRNA) molecules, leading to mRNA degradation or translational repression. Importantly, miRNAs have been demonstrated to modulate various molecular processes by targeting and inhibiting the expression of multiple genes within the same pathway or network, thereby working as master regulators to fine-tune cellular activities (Lin and Gregory, 2015; Peng and Croce, 2016). As miRNA expression is negatively correlated with their target mRNAs, we looked for miRNAs whose expression negatively correlated with oncogenic hubs in UPR-GS in the TCGA database. To this end, the top two miRNAs negatively correlated with oncogenic hubs in UPR-GS belonged to the miR-29 family (miR-29b-2-5p and miR-29c-5p) (Figure 4A). Next, we determined whether these miRNAs can bind/inhibit oncogenic hubs in UPR-GS and found that both miRNAs are predicted to regulate the expression of multiple oncogenic hubs in UPR-GS (Figure 4B). In validation, we found that different gene sets associated with protein folding in the ER and ER stress response are enriched in tumors having low miR-29-GS (comprising of miR-29b-2-5p and miR-29c-5p) scores compared to those having high miR-29-GS scores in bladder cancer patients (Figure 4C; Supplementary Figures S3A–C), suggesting that these miRNAs are indeed involved in regulating UPR in bladder cancer. In addition, genes whose expression is specifically upregulated in Cluster III of bladder cancer are enriched in patients with tumors having low miR-29-GS scores compared to those having high miR-29-GS scores (Figure 4D), whereas genes whose expression is specifically downregulated in Cluster III of bladder cancer are enriched in patients with tumors having low miR-29-GS scores compared to those having high miR-29-GS scores (Figure 4E). Moreover, both miR-29b-2-5p and miR-29c-5p are downregulated in advanced-stage tumors compared to early-stage ones (Figures 4F, G), and high expression of these miRNAs is associated with good survival in bladder cancer patients (Figures 4H, I). Overall, these findings confirm that miR-29b-2-5p and miR-29c-5p are downregulated with cancer progression, and restoration of these miRNAs may inhibit tumor aggressiveness by limiting UPR in bladder cancer.

### 3.4 NFKB inhibits miR-29-GS, drives tumor aggressiveness, and determines poor survival in bladder cancer

**FIGURE 5:**
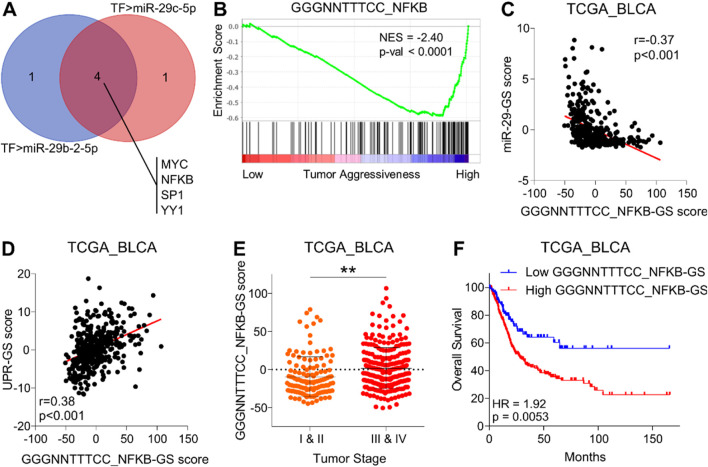
NFKB inhibits miR-29-GS, drives tumor aggressiveness, and determines poor survival in bladder cancer. **(A)** Venn diagram showing intersecting transcription factors predicted to regulate miR-29b-2-5p and miR-29c-5p. **(B)** GSEA showing the enrichment of the GGGNNTTTCC_NFKB gene set between low and high tumor aggressiveness groups in bladder cancer patients from the TCGA database. **(C, D)** A dot plot showing the correlation of GGGNNTTTCC_NFKB-GS with miR-29-GS **(C)** and UPR-GS **(D)** in tumors from bladder cancer patients from the TCGA database. **(E)** A dot plot showing changes in GGGNNTTTCC_NFKB-GS scores between early- and advanced-stage tumors in patients from the TCGA database. **(F)** Kaplan–Meier survival plots showing overall survival analysis based on low and high GGGNNTTTCC_NFKB-GS scores in bladder cancer patients from the TCGA database. TF, transcription factor; BLCA, bladder cancer; NES, normalized enrichment score. **: *p* < 0.01.

Both miR-29b-2 and miR-29c are co-transcribed from the same miR-29b/c gene located at chromosome 1q32.2 (Kriegel et al., 2012). This led us to hypothesize that these miRNAs are potentially downregulated at the transcriptional level during the course of cancer progression in bladder cancer. To test this, we identified common transcription factors (TF) experimentally validated to repress the expression of these miRNAs (Figure 5A). Notably, MYC and NFKB serve as key TFs in this regard. MYC can directly bind to a conserved site 20 kb upstream of miR-29b/c promoter and inhibit miRNA expression (Chang et al., 2008). NFKB-activated Yin Yang 1 (YY1) can bind to miR-29b/c promoter and recruit histone deacetylase 1 (HDAC1) and to EzH2 to repress miRNA expression (Wang et al., 2008). Alternatively, the SP1-NFKB complex can directly recruit HDAC1 to repress the transcription of miR-29b/c (Liu et al., 2010).

Therefore, we aimed to test whether MYC and/or NFKB are associated with progression in bladder cancer. To this end, we performed GSEAs using gene sets related to transcriptional profiles of these TFs and found that NFKB-related gene signatures (NFKB-GS) (Figure 5B; Supplementary Figure S4A), but not those of MYC (Supplementary Figures S4B, C), are enriched in aggressive bladder tumors. In addition, NFKB-GS is negatively correlated with miR-29-GS (Figure 5C; Supplementary Figure S4D) and positively correlated with UPR-GS (Figure 5D; Supplementary Figure S4E) in tumors from bladder cancer patients, suggesting that NFKB may inhibit miR-29-b/c and subsequently upregulate UPR-GS. Notably, NFKB is also predicted to directly regulate the expression of all the genes in UPR-GS in the hTF database (For details, see the Materials and Methods section). Furthermore, NFKB-GS scores are significantly higher in advanced-stage tumors than in early-stage ones (Figure 5E; Supplementary Figure S4F) and are associated with poor survival in bladder cancer patients (Figure 4F; Supplementary Figure S4G), confirming that NFKB is associated with tumor aggressiveness in bladder cancer.

In validation of our findings, NFKB-GS is positively correlated with UPR-GS in bladder cancer patients from GSE13507 (Supplementary Figure S5A) and GSE120736 (Supplementary Figure S5B). A high NFKB-GS score is associated with high-grade tumors compared to low-grade ones in bladder cancer patients from GSE13507 (Supplementary Figure S5C) and GSE120736 (Supplementary Figure S5D). A high NFKB-GS score is associated with advanced-stage tumors compared to early-stage ones in bladder cancer patients from GSE13507 (Supplementary Figure S5E) and GSE120736 (Supplementary Figure S5F). In addition, a high NFKB-GS score is associated with muscle-invasive tumors compared to non-invasive ones in bladder cancer patients from GSE13507 (Supplementary Figure S5G) and GSE120736 (Supplementary Figure S5H). A high NFKB-GS score is associated with disease progression in bladder cancer patients from GSE13507 (Supplementary Figure S5I). Lastly, a high NFKB-GS score is associated with poor overall survival (Supplementary Figure S5J) and poor cancer-specific survival (Supplementary Figure S5K) in bladder cancer patients from GSE13507. Overall, these findings confirm that NFKB inhibits miR-29b/c expression and determines an aggressive disease state and poor survival in bladder cancer patients.

### 3.5 UPR-GS determines the inflammatory tumor environment and fosters tumor aggressiveness in bladder cancer

**FIGURE 6:**
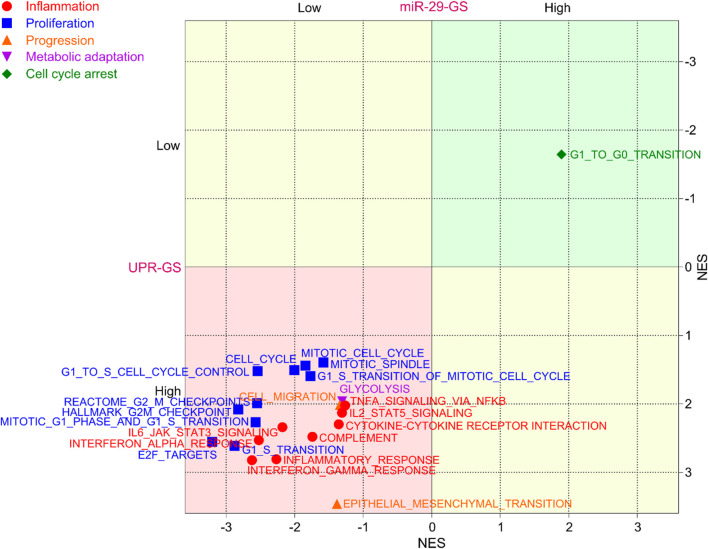
UPR-GS determines inflammatory tumor environment and fosters tumor aggressiveness in bladder cancer. A dot plot showing the overlapping results of GSEA (normalized enrichment score) conducted between low vs. high miR-29-GS score- and between low vs. high UPR-GS score-expressing tumors in bladder cancer patients from the TCGA database, using gene sets related to inflammation, proliferation, tumor progression, metabolic adaptation, and cell cycle arrest.

As NFKB is a key inflammatory TF that regulates the expression of different inflammatory signals and orchestrates inflammatory responses, we tested whether inflammation ensues as a tumor becomes aggressive and found that different inflammation-related gene sets are enriched in aggressive bladder tumors (Supplementary Figure S6A–F). Next, we tested whether NFKB-miR-29b/c axis-regulated UPR-GS is associated with an inflammatory profile of aggressive tumors in bladder cancer patients. We found that different inflammation-related gene sets are enriched in patients with low miR-29-GS and high UPR-GS scores (Figure 6, red). In addition, proliferation-related gene sets attributed to positive regulation of the cell cycle are also enriched in patients with tumors with low miR-29-GS and high UPR-GS scores (Figure 6, Blue). Alternatively, a cell cycle arrest-related gene set is enriched in patients with tumors with high miR-29-GS and low UPR-GS scores (Figure 6, Green). Moreover, tumor progression- (Figure 6, Orange) and metabolic adaptation- (Figure 6, Purple) related gene sets are also enriched in patients with low miR-29-GS and high UPR-GS scores. These findings confirm that NFKB-miR-29b/c axis-driven UPR is a key culprit behind inflammation and tumor aggressiveness in bladder cancer.

## 4 Discussion

The emergence of large-scale omics data has transformed the landscape of cancer research, allowing for in-depth analyses of molecular profiles and the discovery of underlying mechanisms associated with disease (Akhoundova and Rubin, 2022). Moreover, the correlation between clinical characteristics, patient survival, and gene expression facilitates a reverse phenotypic-to-transcriptomic approach, aiding in the exploration of molecular changes and their impact on tumor development, progression, and aggressiveness (Li et al., 2021; Sanguedolce et al., 2022). In our study, we analyzed expression profiling data using stringent molecular + clinical thresholds to unravel the oncogenic targets associated with disease onset, progression, aggressiveness, and patient survival in bladder cancer and identified ER stress response (or UPR) as the key culprit (Figure 1; Supplementary Figure S1). The UPR is a complex cellular pathway crucial for maintaining protein homeostasis within the ER. Due to the proliferative burden, cancer cells often experience increased protein synthesis and folding demands, leading to ER stress and UPR activation as adaptive responses (Oakes, 2020). In particular, we identified an eight-gene UPR-GS comprising TXNDC5, SSR3, RPN1, PDIA5, HSPA5, OSTC, KDELR2, and YIF1B as a key regulator of tumor aggressiveness and poor survival in bladder cancer patients (Figures 2, 3; Supplementary Figure S2). Notably, some of these genes have already been reported for their role in bladder cancer progression. For instance, HSPA5 has been recently shown to serve as a prognostic marker that promotes the proliferation and metastatic spread of the disease by regulating ferroptosis in bladder cancer (Wang et al., 2023), whereas KDELR2 enhances Golgi-mediated secretion to promote bladder cancer growth and metastasis (Meng et al., 2022). Other independent studies have highlighted the role of RPN1, HSPA5, and YIF1B as prognostic markers of the disease along with other sets of genes in bladder cancer (Xu et al., 2021; Jiang et al., 2023; Wang et al., 2024). This is the first instance that TXNDC5 and PDIA5 (genes related to disulfide bond formation-assisted protein folding in ER), SSR3 (a gene related to protein translocation within ER), and OSTC (a gene related to N-linked glycosylation of protein in ER) have been found to be associated with tumor aggressiveness, disease progression, and poor survival in bladder cancer (Figures 2, 3; Supplementary Figure S2).

Previous studies have highlighted aberrant activation of UPR signaling in bladder cancer, aligning with our findings (Yan et al., 2021; Zhang F. et al., 2021). In particular, Zhang et al. explored the role of UPR signaling, identifying a relatively distinct set of UPR-related genes, including CEBPG, HYOU1, IMP3, KDELR3, MTHFD2, PDIA6, POP4, PREB, SRPRB, TATDN2, YIF1A, and ZBTB17, which were associated with survival outcomes and response to chemotherapy and immunotherapy in bladder cancer (Zhang F. et al., 2021). The differences between their findings and ours likely stem from variations in study design, datasets, and analytical methods. Zhang et al. focused on the predictive power of UPR-related genes in therapy response, using a broader dataset including TCGA and GEO datasets (GSE13507, GSE32548, and GSE48075), and employed approaches such as CIBERSORTx for immune infiltration analysis and TCIA for therapy response predictions (Zhang F. et al., 2021). In contrast, our study, using the TCGA database as a sole discovery dataset, focused on understanding the molecular mechanisms underlying disease onset, progression, and patient survival, identifying elevated ER stress response as a key driver of these processes. Despite these methodological differences, both studies highlight the interplay between UPR activation and inflammation, with Zhang et al. exploring its role in immune evasion and therapy resistance (Zhang F. et al., 2021), while our work linking it to inflammatory stress as a driver of tumor aggressiveness in bladder cancer (Figures 5, 6).

Inflammation plays a dual role in cancer, acting as both a driver of tumorigenesis and a promoter of tumor progression (Crusz and Balkwill, 2015; Zhao et al., 2021). Activation of NFKB, a key regulator of inflammation, can result from various stimuli, including cytokines, growth factors, and cellular stressors, leading to the expression of genes involved in cell survival, proliferation, and inflammation. This pro-inflammatory microenvironment not only fuels tumor growth but also contributes to therapy resistance and immune evasion (Zhang T. et al., 2021). Inflammation, in general, and the NFKB pathway, in particular, are frequently activated in bladder cancer cells, communicating with different signaling pathways through transcriptional repertoire (Mukherjee et al., 2015). This aligns with our findings that NFKB-related gene sets are enriched in advanced-stage, aggressive bladder tumors and regulate UPR by suppressing miR-29b/c expression ((Figure 5; Supplementary Figures S4–S6). miRNAs from the miR-29 family have been established as tumor suppressor miRNAs (often downregulated in tumor tissues), regulating a diverse range of molecular mechanisms, including proteostasis, metabolism, proliferation, apoptosis, EMT, metastasis, fibrosis, angiogenesis, and immune-modulation by targeting plethora of mRNA targets (Kwon et al., 2019; Horita et al., 2021). In line with this, we observed that miR-29b/c, in particular, are downregulated in bladder cancer and have the potential to limit tumor aggressiveness by targeting UPR-GS (Figure 4; Supplementary Figure S3) which was predicted as a target of miR-29b-2-5p and miR-29c-5p. Upstream, transcription from miR-29b/c is reported to be primarily suppressed by either MYC or NFKB transcription factor (Chang et al., 2008; Wang et al., 2008), which we sorted out to be NFKB in the case of bladder cancer, as NFKB-related, but not MYC-related, gene sets are enriched in advanced-stage, aggressive tumors in bladder cancer patients (Figure 5; Supplementary Figure S5).

Aberrant activation of UPR signaling is also intertwined with the inflammatory tumor microenvironment as it has been associated with immune cell infiltration (Yan et al., 2021; Zhang F. et al., 2021), creating a complex interplay between inflammation and UPR. We also observed that UPR-GS is a biomarker of inflammation, proliferation, tumor progression, and metabolic adaptation in bladder cancer (Figure 6). This hints that UPR plays a much more important role than a mere downstream effector of inflammation, influencing bladder cancer’s molecular landscape and clinical outcomes as observed. This can be true because of the generalized role of UPR in maintaining cellular homeostasis and fueling a plethora of survival mechanisms by ensuring proteostasis in actively proliferating cancer cells (Oakes, 2017).

**FIGURE 7:**
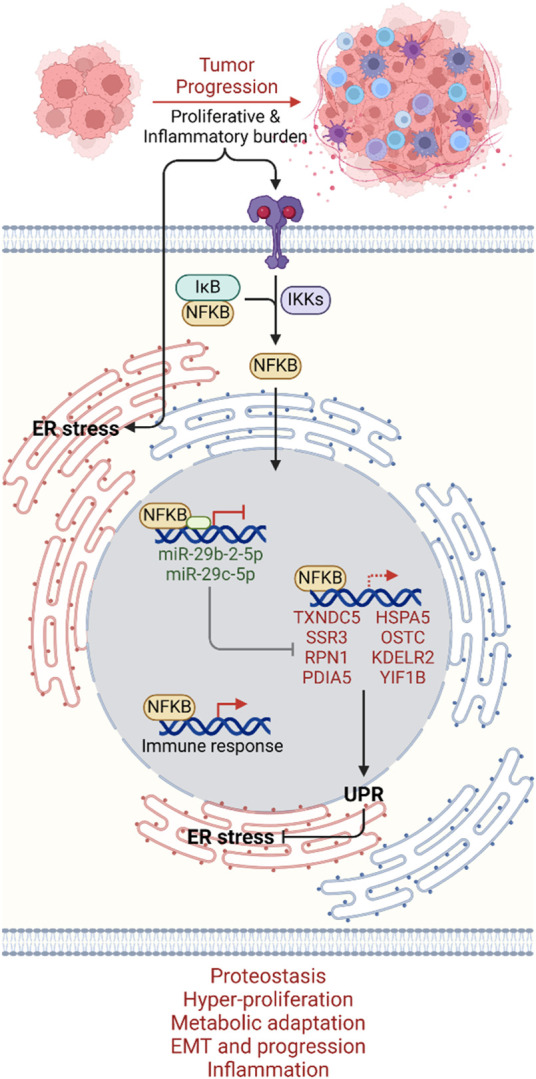
Mechanistic summary. As tumors progress to an aggressive state, proliferative and inflammatory burdens induce ER stress on the one hand and inflammatory signal-driven activation of the NFKB transcription factor on the other hand. NFKB, upon translocating to the nucleus, promotes UPR by suppressing miR-29-2-5p and miR-29c-5p transcription, which can no longer inhibit UPR-GS. NFKB may also directly regulate UPR-GS along with its signature immune responses. UPR-GS actively limits ER stress, ensuring proteostasis and promoting hyper-proliferation, metabolic adaptation, EMT, cancer progression, and inflammation in bladder cancer. Dim, Under-active axes; Bright, Hyperactive axes.

Future investigations in bladder cancer research are poised to delve deeper into the intricate molecular networks governing ER stress responses and inflammation. Here, we provided a promising avenue for the exploration of targeted therapies that modulate the NFKB-miR-29b/c axis to attenuate UPR-GS activation and mitigate tumor aggressiveness (Figure 7), though *in vitro* and *in vivo* validation of our findings are warranted. Integrating multi-omics data, including genomics, transcriptomics, proteomics, and metabolomics, coupled with machine learning and artificial intelligence, can enable the development of personalized treatment strategies (Jendoubi, 2021; Quazi, 2022). Collaborative efforts across disciplines, translational research platforms, and clinical trials will be pivotal in translating our findings into clinical practice (Castaneda et al., 2023), ultimately improving the management of bladder cancer patients by restoring miR-29b/c expression and subsequently suppressing UPR, thereby mitigating tumor aggressiveness and limiting bladder cancer burden.

## 5 Conclusion

Overall, this study highlights the central role of UPR in driving tumor aggressiveness and poor patient survival in bladder cancer. As tumors progress to an aggressive state, the proliferative and inflammatory burdens induce ER stress and activate NFKB. NFKB promotes UPR by suppressing miR-29-2-5p and miR-29c-5p transcription, which can no longer inhibit UPR-GS. Downregulation of miR-29b/c downstream of NFKB, associated with elevated UPR-GS, suggests a potential avenue for limiting tumor aggressiveness and improving patient outcomes. Targeting the UPR pathway and modulating miR-29b/c expression could offer novel therapeutic strategies. Additionally, UPR-GS and miR-29b/c levels may serve as valuable prognostic markers, aiding in personalized treatment approaches and prognostic assessments in bladder cancer management.

## Data Availability

The original contributions presented in the study are included in the article/Supplementary Material; further inquiries can be directed to the corresponding authors.
