# Supplementary material for: An unfolded protein response (UPR)-signature regulated by the NFKB–miR-29b/c axis fosters tumor aggressiveness and poor survival in bladder cancer
Source: Front Mol Biosci. 2025 Feb 14;12:1542650. doi: 10.3389/fmolb.2025.1542650 (PMC11867963; doi:10.3389/fmolb.2025.1542650)
Supplement: Supplementary file 2 [file DataSheet1.pdf]

An unfolded protein response (UPR)-signature regulated by NFKB-miR-29b/c axis fosters tumor aggressiveness and poor survival in bladder cancer

Supplementary Table

Table S1: List of gene sets used in the study for patient classification or gene set enrichment analysis (GSEA).

Table S2: Gene set scores calculated in the study for patients from TCGA database, and GEO datasets (GS13507 and GSE120736).

Supplementary Figures

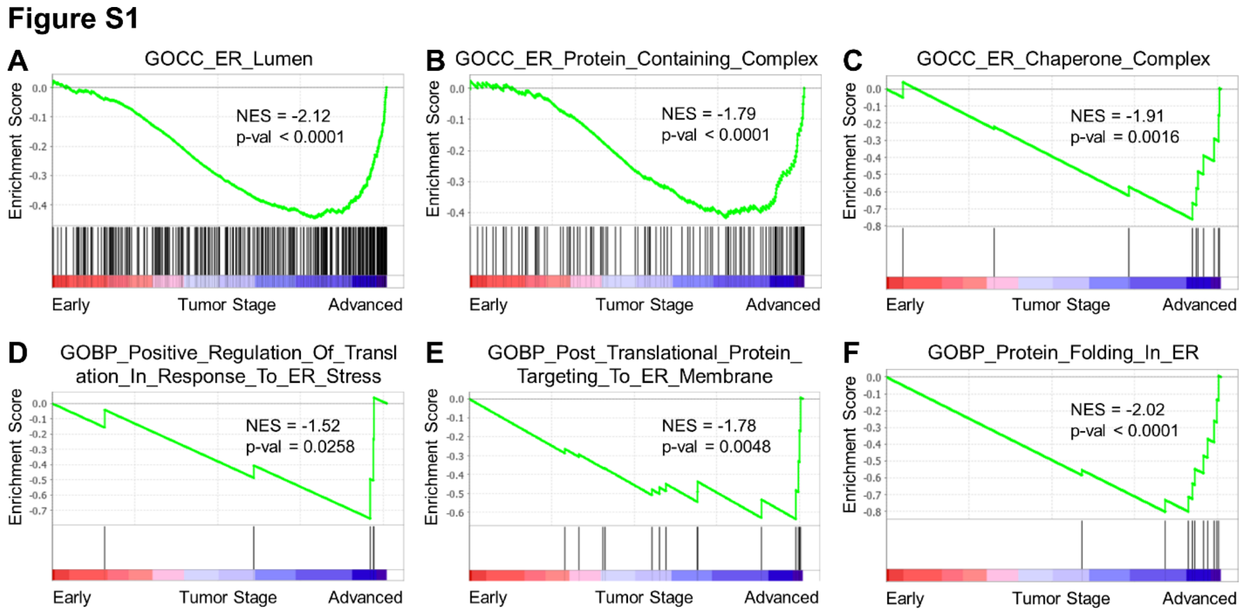

Figure S1: ER stress-related gene sets are associated with aggressive disease state in bladder cancer. (A-F) GSEA showing the enrichment of ER stress-related gene sets, GOCC_ER_Lumen (A), GOCC_ER_Protein_Containing_Complex (B), GOCC_ER_Chaperone_complex (C), GOBP_Positive_regulation_Of_Translation_in_Response_To_ER_Stress (D), GOBP_Post_Translational_Protein_Targeting_To_ER_Membrane (E), and GOBP_Protein_Folding_In_ER (F), between early and advanced stage tumors in bladder cancer patients from TCGA database. NES: normalized enrichment score.

Figure S2

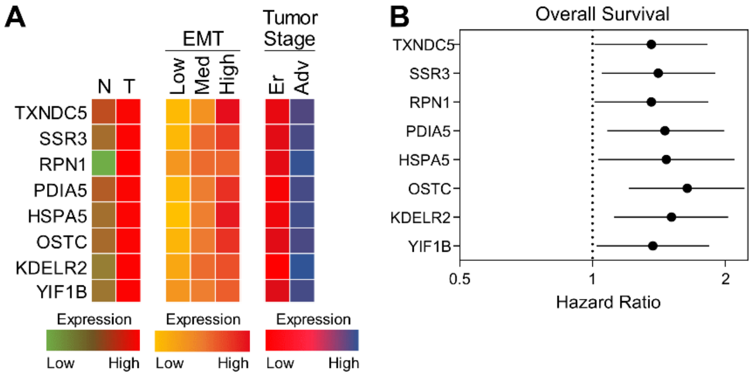

Figure S2: UPR-related oncogenic hubs are associated with tumor onset, disease progression and poor survival in bladder cancer. (A) Heatmaps showing expression change in UPR-related oncogenic hubs between normal and tumor tissues (left), among EMT score based tumor aggressiveness groups (low, intermediate, high) (middle), and between early and advanced stage tumors (right) in bladder cancer patients from TCGA database. (B) Forest plot showing survival association of UPR-related oncogenic hubs in bladder cancer patients from TCGA database.

Figure S3

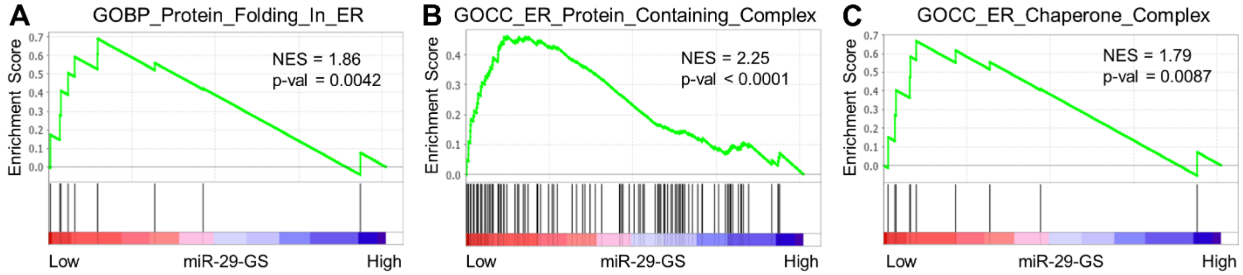

Figure S3: ER-stress related gene sets are associated with low miR-29-GS in bladder cancer. (A-C) GSEA showing the enrichment of ER stress-related gene sets, GOBP_Protein_Folding_In_ER (A), GOCC_ER_Protein_Containing_Complex (B), and GOCC_ER_Chaperone_complex (C), between low and high miR-29-GS expressing tumors in bladder cancer patients from TCGA database. NES: normalized enrichment score.

Figure S4

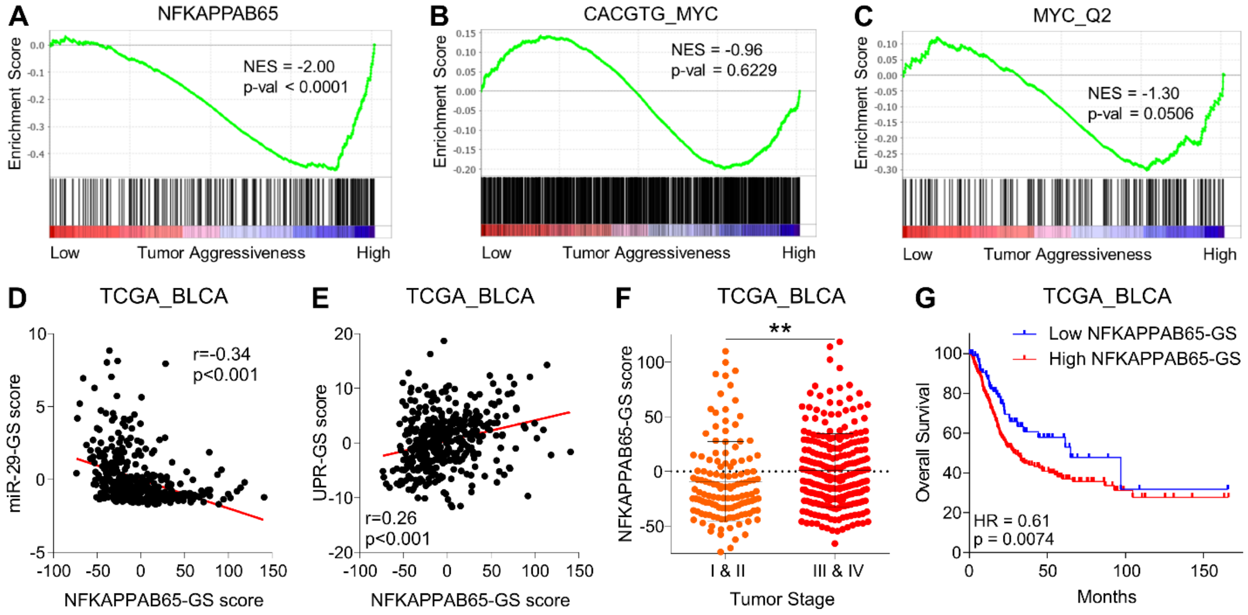

Figure S4: NFKB, not MYC, is associated with aggressive disease state and poor survival in bladder cancer. (A-C) GSEA showing the enrichment of gene sets, NFKAPPAB65 (A), CACGTG_MYC (B), and MYC_Q2 (C), between low and high tumor aggressiveness groups in bladder cancer patients from TCGA database**.** (D and E) Dot-plot showing correlation of NFKAPPAB65-GS with miR-29-GS (D) and UPR-GS (E) in tumors from bladder cancer patients from TCGA database. (F) Dot-plot showing changes in NFKAPPAB65-GS score between early and advanced stage tumors in patients from TCGA database. (G) Kaplan-Meier survival plots showing overall survival analysis based on low and high NFKAPPAB65-GS score in bladder cancer patients from TCGA database. BLCA: bladder cancer, NES: normalized enrichment score. **: p<0.01.

Figure S5

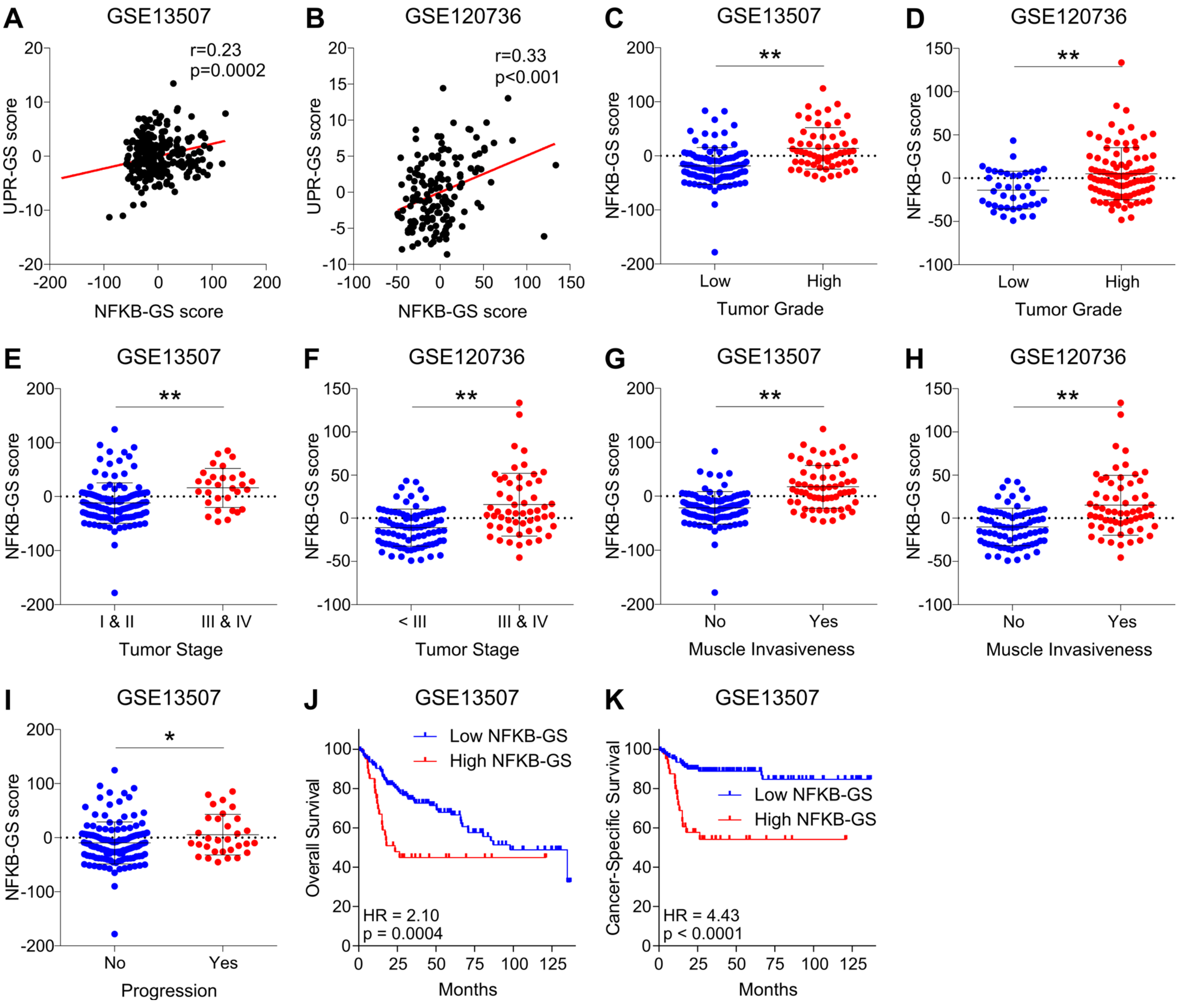

Figure S5: NFKB-GS is associated with aggressive disease state and poor survival in bladder cancer. (A and B) Dot-plot showing correlation of NFKB-GS with UPR-GS in tumors from bladder cancer patients from GSE13507 (A) and GSE120736 (B). (C and D) Dot-plots showing changes in NFKB-GS score between low and high grade tumor in bladder cancer patients from GSE13507 (C) and GSE120736 (D). (E and F) Dot-plots showing changes in NFKB-GS score between early and advanced stage tumors in bladder cancer patients from GSE13507 (E) and GSE120736 (F). (G and H) Dot-plots showing changes in NFKB-GS score between non-muscle invasive and muscle invasive subtypes of tumors in bladder cancer patients from GSE13507 (G) and GSE120736 (H). (I) Dot-plot showing changes in NFKB-GS score with tumor progression in bladder cancer patients from GSE13507. (J and K) Kaplan-Meier survival plots showing overall survival (J) and cancer-specific survival (K) analysis based on low and high NFKB-GS score in bladder cancer patients from GSE13507 dataset. **: $p<0.01$, *: $p<0.05$.

Figure S6

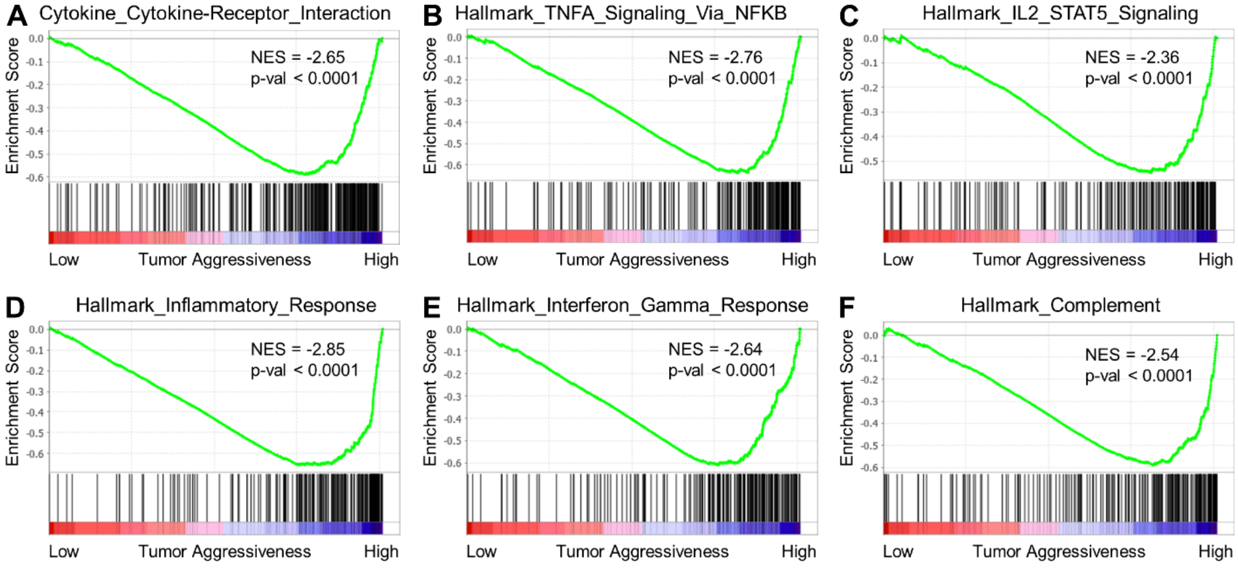

Figure S6: Inflammation-related gene sets are associated with tumor aggressiveness in bladder cancer. (A-F) GSEA showing the enrichment of inflammation-related gene sets, Cytokine_Cytokine_Receptor_Interaction (A), Hallmark_TNFA_Signaling_Via_NFKB (B), Hallmark_IL2_STAT5_Signaling (C), Hallmark_Inflammatory_Response (D), Hallmark_Interferon_Gamma_Response (E), and Hallamrk_Complement (F), between low and high tumor aggressiveness groups in bladder cancer patients from TCGA database. NES: normalized enrichment score.
